# Evaluation of a minimally invasive glucose biosensor for continuous tissue monitoring

**DOI:** 10.1007/s00216-016-9961-6

**Published:** 2016-10-15

**Authors:** Sanjiv Sharma, Zhenyi Huang, Michelle Rogers, Martyn Boutelle, Anthony E. G. Cass

**Affiliations:** 1 Department of Chemistry & Institute of Biomedical Engineering, Imperial College, South Kensington Campus Exhibition Road, London, SW7 2AY UK; 2 Department of Bioengineering, Imperial College, South Kensington Campus, London, SW7 2AZ UK

**Keywords:** Microneedles, Electrochemical sensor, Continuous glucose monitoring (CGM)

## Abstract

We describe here a minimally invasive glucose biosensor based on a microneedle array electrode fabricated from an epoxy-based negative photoresist (SU8 50) and designed for continuous measurement in the dermal compartment with minimal pain. These minimally invasive, continuous monitoring sensor devices (MICoMS) were produced by casting the structures in SU8 50, crosslinking and then metallising them with platinum or silver to obtain the working and reference electrodes, respectively. The metallised microneedle array electrodes were subsequently functionalised by entrapping glucose oxidase in electropolymerised polyphenol (PP) film. Sensor performance in vitro showed that glucose concentrations down to 0.5 mM could be measured with a response times (T_90_) of 15 s. The effect of sterilisation by Co60 irradiation was evaluated. In preparation for further clinical studies, these sensors were tested in vivo in a healthy volunteer for a period of 3–6 h. The sensor currents were compared against point measurements obtained with a commercial capillary blood glucometer. The epoxy MICoMS devices showed currents values that could be correlated with these.

**Graphical Abstract:**
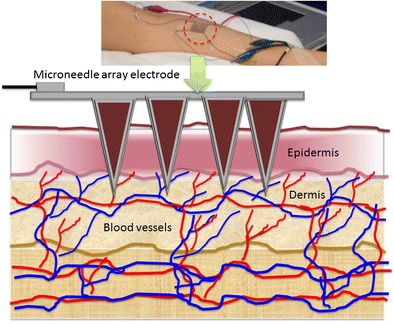
Microneedle arrays for continuous glucose monitoring in dermal interstitial fluid

Microneedle arrays for continuous glucose monitoring in dermal interstitial fluid

## Introduction

First approved by the FDA for the management of diabetes in 1999, continuous glucose monitoring (CGM) technology has now emerged as an important diagnostic tool for detecting episodes of high blood glucose (hyperglycaemia) and low blood glucose (hypoglycaemia). The real time information on changes in glucose concentrations in terms of the direction, duration and magnitude provided by CGM devices improves glycaemic control as assessed by a reduction in the levels of glycated haemoglobin (HbA1c) [1]. There are reports that suggest that CGM is also associated with reduction in episodes of hypoglycaemia in both children and adults with type 1 diabetes^1^. The advantages of regular use of CGM have been quantified; for instance, for every 1-day increase in sensor usage per week, an average HbA1c reduction of 0.15 % is observed [2, 3]. However, despite the clear benefits of continuous glucose monitoring, it has not been widely implemented in the routine management of type 1 diabetes (T1D) due to several major challenges. This is evident from T1D exchange data demonstrating that CGM technology is being used by only 6.5 % of people with type 1 diabetes in the USA (even with reimbursement), and that among those individuals who have used a CGM, two thirds subsequently stopped using it [4–6]. The major disadvantages associated with the commercially available CGM devices are their invasiveness and the associated discomfort, negatively affecting adherence and the subsequent effectiveness of CGM. Data on CGM sensor accuracy have shown clinically significant, reduced accuracy in the critical hypoglycaemic range representing another important barrier to use with a high frequency of false hypoglycaemia alarms [7]. Finally, variable reimbursement for existing CGM systems limits access and high costs limit self-funding opportunities for people with diabetes [8]. We have sought to address these limitations through the development of a novel electrochemical sensor based on microneedle array electrodes (MICoMS) for CGM. Earlier, we reported on the use of microneedle structures functionalised with GOx, a TTF mediator and an epoxy PU membrane for the measurement of glucose in vitro [9, 10]. However, these were unsuited for in vivo use and hence this paper describes the development of minimally invasive, continuous monitoring sensors (MICoMS) suitable for measurement in the dermal interstitial fluid.

Fabrication of microneedles for transdermal drug and vaccine delivery has been reported extensively in the literature [11–16]. The microneedle array electrochemical sensors reported here have the potential for mass production by techniques such as injection moulding and hot embossing and thus lowering costs so they need be used for only short periods (24–48 h) before replacement. This should minimise problems associated with long-term implantation.

**Fig. 1:**
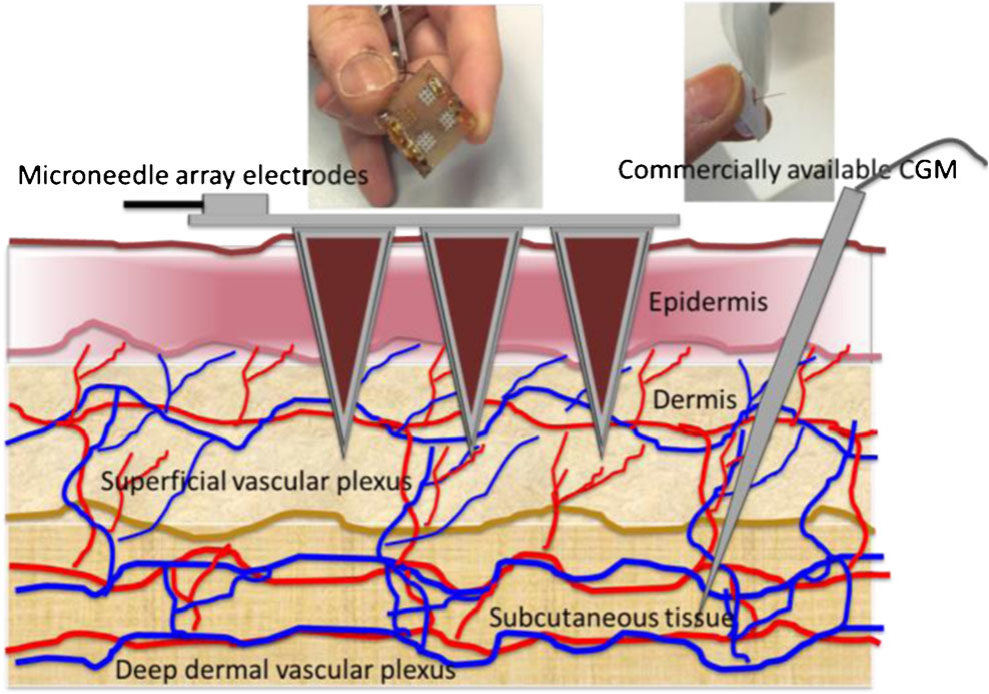
Showing the two different approaches (microneedle array electrodes versus commercial CGM) and the two regions of the skin compartment used to access the interstitial fluid

Some of the approved commercial devices for continuous glucose monitoring include Enlite (Medtronic), G5 (Dexcom) and the FreeStyle Libre (Abbott). Compared to the conventional subcutaneous microneedle-based sensors, the MICoMs demonstrated in this paper offer following major advantages: (1) By virtue of their dimensions, they are minimally invasive and can be easily inserted and regularly replaced as compared to subcutaneous devices, which require implantation using an applicator. (2) Sampling mainly in the dermal space (Fig. 1) will lead to reduced biofouling effects [17]. (3) It provides a larger electrode surface area and thus larger currents (>100 nA) than other subcutaneously implanted sensors (>10 nA). (4) The microneedle arrays electrodes (MICoMS) can be modified and distributed into working electrodes and reference electrode facilitating multiplexing or single analyte sensing by multiple MICoMSs. The continuous glucose biosensors described here are first generation glucose sensors made of platinum functionalised by glucose oxidase with electropolymerised phenols.

## Materials and methods

### Materials

SU-8 photoresist was obtained from Chestech Ltd, UK. Ferrocene carboxylic acid (FCA), phenol was obtained from Sigma-Aldrich, UK. Glucose oxidase (Glucose Oxidase HPS300, Activity 239 U/mg powder @25oC, source Ex *Aspergillus niger*) was procured from Sekisui Diagnostics (UK), Ltd. For the in vivo studies comparison, an ACCU-CHEK Aviva blood glucose system was used for intermittent capillary blood.

### Fabrication of the microneedle array glucose sensor

**Fig. 2:**
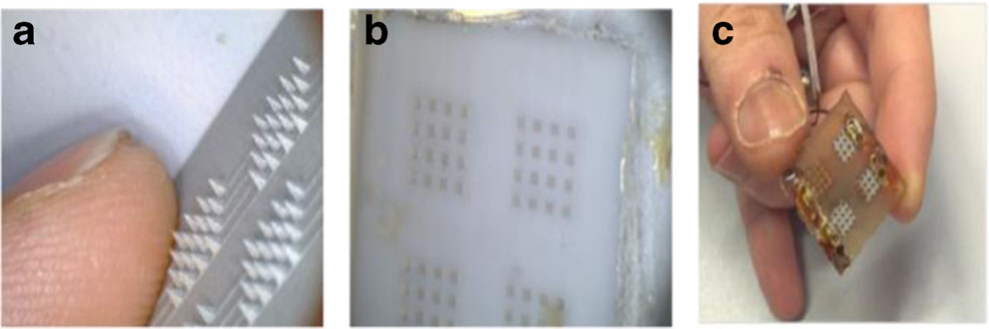
Fabrication procedure of microneedle array electrode. **a** Aluminium master of microneedle array made by EDM; **b** PDMS mould obtained from the aluminium master; **c** bare microneedle array electrodes with one reference and three working electrode arrays

Aluminium masters, fabricated using an electrical discharge machining (EDM) technique, were used to create moulds of polydimethoxy siloxane (PDMS). The PDMS moulds were subsequently used to cast SU8 50 using vacuum and spinning. The SU8 50 cast PDMS moulds were subjected to 10 mmHg vacuum pressure for 2 min and spun in petri dish at 4000 rpm for 30 min in a centrifuge (Eppendorf). SU8 50 was then cross-linked by exposure to UV light at 365 nm for 30 min. The final epoxy MICoMSs were then obtained by peeling off from the PDMS layer (Fig. 2). Using the casting moulding technique, >80 epoxy microneedle arrays can be fabricated per week.

### Characterisation of microneedle devices using scanning electron microscopy (SEM)

The microneedle array electrode (MICoMS)-based continuous glucose sensor consists of a three-dimensional out-of-plane microneedle array, with 64 microneedles perpendicular to the base plate and arranged as four 4 × 4 arrays. The microneedle array electrode-based devices were characterised using a JEOL 5610 SEM. Attempts to imaging the SU8 50 microneedles in vivo using OCT were unsuccessful due to high absorbance of the material and therefore optically transparent polycarbonate structures were used instead.

### Metallisation, modification of microstructure region and wire bonding to device

One of the microneedle array was masked with a tape and the remaining three coated with an adhesion layer of titanium (15 nm) followed by a platinum layer (50 nm) by conformal sputtering (DC sputter system, JLS MPS 500) in a clean room to produce three electrodes. In the next step, these three electrodes are masked and the remaining region metallised with silver (100 nm). In this work, the three platinum microneedle array electrodes are used as working electrodes and the fourth, metallised with silver, was treated with a saturated FeCl_3_ solution to obtain a Ag/AgCl reference electrode. The potential of the integrated reference electrode was measured against an external reference electrode and found to be higher by 30 ± 4 mV.

One millimetre holes were drilled at the periphery of the metallised part of microneedle structures, using a bench top drill. Wire bonding was done using a wrap equipment wire (RS Pro Black, Kynar Wire Wrap Equipment Wire, 0.05 mm^2^ CSA, 300 V 0.4 A, 50 m) run through the holes and held in place by silver conductive, adhesive paint (RS Silver Bottle Paint Conductive Adhesive) and ATACS5104 (Sigma) epoxy adhesive.

The electrode surface of the microneedle array was assessed by cyclic voltammetry (CV) using ferrocenecarboxylic acid (FCA) as a redox probe. In addition, cyclic voltammetry and chronoamperometry were performed to model the behaviour of the microneedle array electrode.

### Functionalisation of the microneedle array electrode by electropolymerisation

The glucose biosensors described here were functionalised with an enzyme film using an electropolymerisation method described earlier [1]. For the electropolymerised polyphenol, the electrode was placed in a 50 mM solution of phenol and 10 mg/mL of GOx enzyme dissolved in 100 mM PBS. The pH of the solution was buffered to pH 7.2. Each cycle comprised of holding the working electrode at 0 V for 20 s, polarising it to a potential of 0.9 V for 15 min for electropolymerisation of the film. Each cycle was repeated six times to obtain the desired film thickness and glucose oxidase loading. Following electropolymerisation, the biosensor was gently rinsed with de-ionised water and stored dry overnight at 4 °C before use.

### Evaluation of GOx functionalised microneedle arrays

#### Dose–response curves of functionalised microneedle array electrodes

Electrochemical measurements were performed in a two-electrode configuration with CHI 1030b potentiostat (CHI Instruments) running general-purpose electrochemical software (GPES v4). To make maximum use of the platinum microneedle arrays as working electrodes, a two electrode was used. With the currents passed, there is no evidence to suggest that RE has changed its performance.

Two hundred microliters aliquots of varying glucose concentrations prepared in 100 mM PBS solution (pH 7.2) were added stepwise to the devices with the microneedle electrode arrays polarised at 0.7 V against the monolithically integrated Ag/AgCl counter/reference electrode. The steady-state current measured at 60 s was determined as a function of the glucose concentration over the range 0 to 30 mM. The apparent *I*_max_ and *K*_m_ were found by plotting from the current at 60 s against concentration and fitting the points with the Michealis-Menten equation fitting in OriginPro 9.0. The Michealis-Menten equation is given as:

$$ I={I}_{\max}\left[S\right]/{K}_{\mathrm{m}}+\left[S\right] $$

where *I*_max_ is the maximum current, [*S*] is the glucose concentration and *K*_m_ is the Michaelis constant for glucose oxidase.

#### Chemical crosstalk studies

There is a possible complicating factor in any sensor array based on the detection of diffusible species. If the sensors are too close together, hydrogen peroxide generated on one electrode diffuses to another. To confirm the absence of sensor ‘chemical crosstalk’, one set of the microneedle electrodes was functionalised with glucose oxidase as described above. The other two-microneedle sets of electrodes on the same device were not functionalised.

#### Dose–response curves post sterilisation of functionalised microneedle array electrodes

The microneedle arrays were subjected to 25 kGy of Co60 (Synergy Health) for assay of the bio burden levels on the microneedle arrays and subsequent estimation of dose for sterilisation. These studies were done in accordance with ISO 11137-2:2012, Sterilisation of Health Care Products-Radiation-Part2: Establishing the Sterilisation dose.

### In vivo studies using the microneedle array electrodes-based glucose biosensors

Sterilised microneedle array electrode-based glucose biosensors were inserted into the forearm of a healthy volunteer and their glucose concentration measured every 15 min using a finger stick glucometer. A CHI potentiostat was used to continuously bias the inserted microneedle array working electrodes at 0.7 V against the integrated counter/reference electrode with a data point being collected every 60 s. The in vivo studies were made on two separate occasions each lasting 3–6 h.

For the in vivo studies in consented healthy volunteer, approvals were obtained from the research ethics committee (REC reference: 16/LO/0007, IRAS project ID 190530).

## Results and discussion

### SEM characterisation of base microneedle array structures

**Fig. 3:**
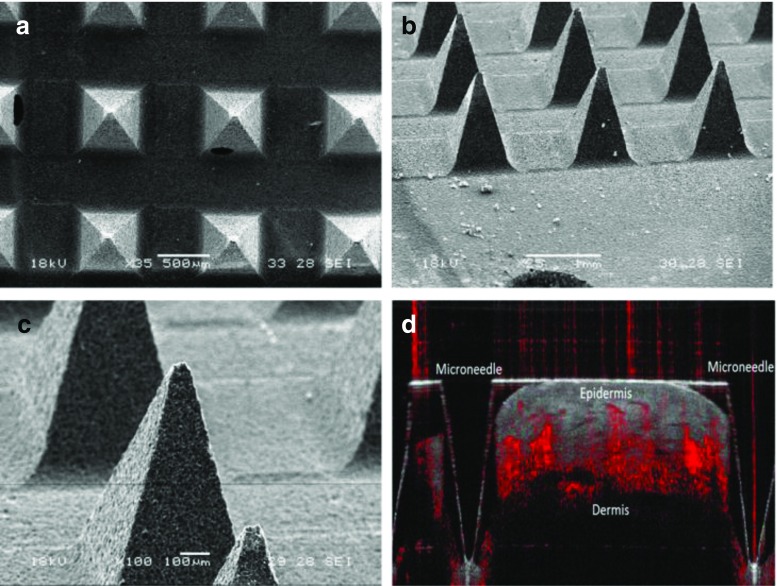
Showing SEM and OCT image(s) of the bare microneedles. *3(a)*, Top image showing the micrographs at ×35 magnification; *3(b), (c)* showing SEM micrographs obtained after tilting the sample stage at 60° and at ×25 and ×100. *3(d)* shows an OCT image of a transparent microneedle of similar geometry in a healthy volunteer

SEM images of the base microneedle arrays at different magnifications and tilt angles revealed the dimensions of each microneedle. The microneedles were ≈1000 μm in length with a base of 600 μm, a tip diameter of 35 μm and pitch of 1200 μm (tip-to-tip distance). OCT image obtained after insertion of the sterilised, uncoated microneedle fabricated from polycarbonate material is shown below in Fig. 3(d).

### Modelling the electrochemical behaviour of microneedle array electrodes

Since there is no cited work on the diffusion profile of pyramidal microneedle array electrodes, the microneedle array electrode was electrochemically characterised with a simple assumption. It was assumed that each 4 × 4 microneedle array behaves as a planar electrode. The diffusion current for a planar electrode in a chronoamperometry measurement is described in Cottrell equation below:

$$ {i}_d(t)=nFA{D}_o{C^{*}}_o/{\left(\pi {D}_ot\right)}^{1/2} $$

where *A* is the area of the electrode, *F* = Faraday’s constant (96,485 C mol^−1^), *D*_*o*_ the diffusion coefficient of ferrocene carboxylic acid (5.7 × 10–6 cm^2^ s^−1^), *n* the number of electron(s) involved (*n* = 1), *t* time in seconds and *C* ^*^_o_ the concentration of ferrocene carboxylic acid (5 mM).

**Fig. 4:**
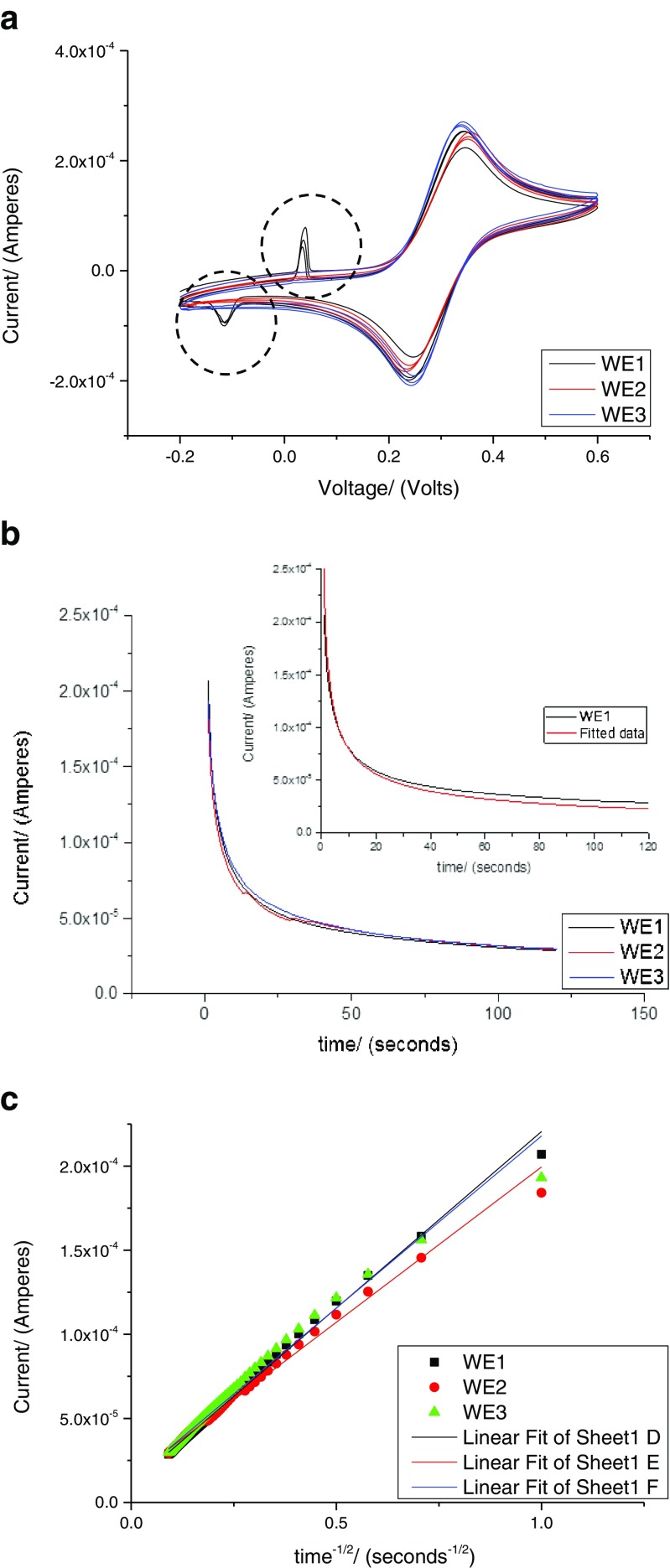
**a** Cyclic voltammetry of 200 μL of 5 mM FCA at a scan rate of 100 mV s^−1^. The artefacts (*dotted circles*) at *V* < 0.1 V seen for WE1 is due to epoxy used for wire bonding. **b** Chronoamperometry measurement with 200 μL of 5 mM ferrocene carboxylic acid with the *inset* showing the fitted data. **c**: *I v*/*s t* ^−1/2^ plot obtained from chronoamperometry measurement with 200 μL of 5 mM ferrocene carboxylic acid

The area of the microneedle array was evaluated by chronoamperometry and the details of the steps are described as following. Cyclic voltammetry with 5 mM ferrocene carboxylic acid redox probe was performed to find the peak potential *E*_P_ (forward) which was 0.38 V (Fig. 4). After applying a potential of 0.50 V (*E*_P_) to the electrode with 200 μL of the 5 mM ferrocene carboxylic acid, the current was measured for 120 s (Fig. 4b).

Most of the current decays within the first 10 s and this current, *i*_*d*_, was plotted against *t* ^-1/2^, as shown in Fig. 4c, with the slope *nFAD*_*o*_ *C* ^*^_o_ / (*πD*_*o*_)^1/2^. At short period of time, the Cottrell term *nFAD*_*o*_ *C* ^*^_o_ */* (*πD*_*o*_)^1/2^ dominates, and for longer period of time, it is anticipated that other factors such as convection come into play.

As seen from Fig. 4b, inset, fitted current values are higher than the experimental values at shorter time and lower at longer time. This discrepancy (approx. 3 %) is maintained throughout the later part of the 120-s measurements. At longer times, the noise is significantly lower than one would expect for a simple planar electrode. It appears that the microneedle structures may act as pillars and so stabilise the current.

The area of equivalent microneedle array electrodes (WE1, 2 and 3) were calculated from the slope of the Cottrell plot (Fig. 4c). The area of the microneedle array electrode calculated using the Cottrell equation, for WE1, 2 and 3 were 0.32, 0.28 and 0.31 cm^2^, respectively, which is in close agreement to the value calculated geometrically (0.38 cm^2^) (Table 1). The area of each microneedle array was calculated using the following values: area of microneedle array (0.5 × 0.5) cm^2^, surface area of 16 pyramidal microneedles (each with base 0.06 cm, height 0.1 cm).

**Table 1:** Intercept and slope obtained from the Cottrell plots for microneedle array electrodes

Electrode	Intercept	Slope	Statistics	Area of electrode (in cm^2^)	
	(Amperes)	(Amperes)	Adj. *R*-square	Experimentally	Geometrically
WE1	1.075E−05 + 0.027	2.10E−04 + 0.013	0.99559	0.32	0.38
WE2	1.49E−05 + 0.034	1.85E−04 + 0.016	0.99109	0.28	0.38
WE3	1.075E−05 + 0.05	2.05E−04 + 0.024	0.98378	0.31	0.38

Values are ± standard error

These studies provide valuable insight on the behaviour of the microneedle array electrodes in context to devices for continuous glucose sensing in the interstitial fluid. As seen from the OCT images with polycarbonate microneedles (penetration depth 800 μm), we anticipate that 80 % of the microneedle array length (from the tip) will be in contact with the ISF and the mass transport will be mainly driven by diffusion of the glucose to the sensor surface.

### Evaluation of GOx functionalised microneedle array electrodes

#### Dose–response curves of microneedle array electrodes functionalised by phenol electropolymerisation

**Fig. 5:**
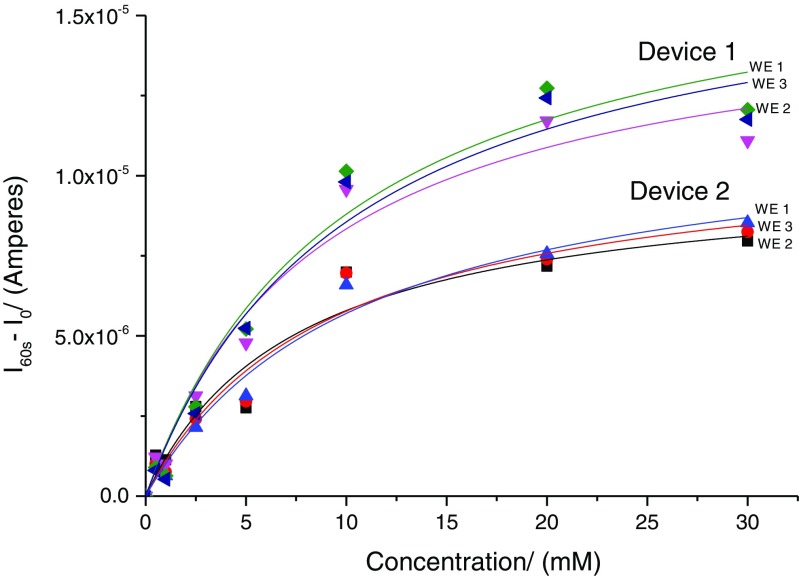
Dose–response curves obtained for two devices functionalised with polyphenol films entrapped with glucose oxidase. Data is current values at 60 s minus background current

The electropolymerised devices were tested with various glucose concentrations using chronoamperometry (Fig. 5).

From the chronoamperometry studies, the dose–response curves were obtained from filling to the Michealis-Menten equation. It is observed that glucose concentration as low as 0.5 mM could be easily measured. The average 90 % response time T_90_ was approximately 15 ± 2 s. The responsiveness, *R*, of each sensor was also calculated with equation below,

$$ R={I}_{10\mathrm{m}\mathrm{M}}-{I}_{1\mathrm{m}\mathrm{M}}/9 $$

where *I*_10mM_ and *I*_1mM_ were the steady-state currents at glucose concentration of 10 and 1 mM, respectively.

In total six devices were fabricated and the average *K*_m_ values for all the sensor readings (*n* = 50) was 9.87 + 1.62 mM. Variations in *K*_m_ value between the devices were seen to be larger than that between the sensors within a device. The devices showed variability in the *I*_max_ between the MICoMS electrodes. The plausible reasons for this variation between the electrodes in the same device are factors such as differences in area of the MICoMS electrodes, distance of the working electrode from the reference electrode yielding variable IR drops and the total enzyme loading during electropolymerisation.

The variations in the area of the MICoMS electrodes are probably introduced during the masking step. In general, it is found that two of the electrodes give more similar current response compared to the third and this appears to correlate with the distance from the integrated reference electrode. Herein, two electrodes that agree are equidistant and the outlier slightly further away. Finally, the It curves obtained from the electro polymerisation of phenol to yield polyphenol films entrapped with GOx enzyme are different for the three MICoMS electrodes suggesting differences in enzyme loading. Normalisation of the current values with the total charge obtained from the It curves from electropolymerisation reduces the variation between the microneedle arrays.

#### Chemical crosstalk studies

**Fig. 6:**
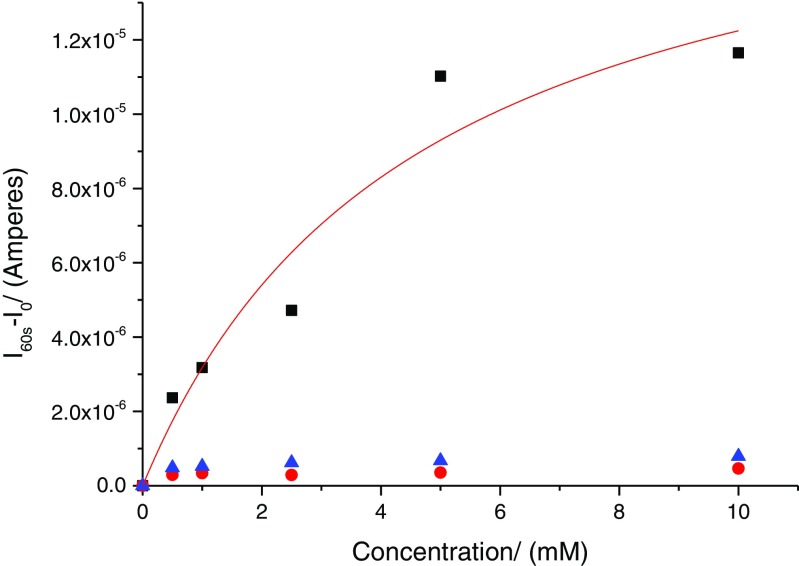
Dose–response curves obtained after functionalisation of a single working electrode (WE1—*black squares*) with glucose oxidase (*K*_m_ = 4.6 ± 2.29 mM, *I*_max_ = 180 ± 4 μA). WE2 (*blue triangles*) and WE3 (*red circles*) are blank electrodes

To confirm the absence of sensor ‘chemical crosstalk’, one of the microneedle electrodes was functionalised with glucose oxidase as described above. The other two were left unfunctionalised. As seen in Fig. 6, no crosstalk between the microneedle electrodes is observed.

#### Comparison of pre- and post sterilisation dose–response curves of functionalised microneedle array electrodes

**Fig. 7:**
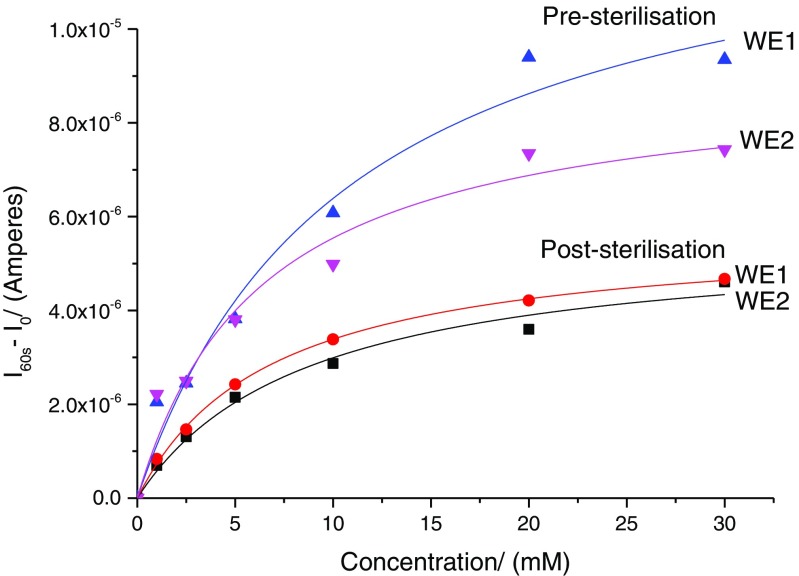
Dose–response curves for devices, pre and post sterilisation. As seen here, the *I*_m_ values drop

There are reports in the literature suggesting that sterilisation of glucose biosensors with gamma irradiation from 25 to 35 kGy adversely affect performance [18]. Dose–response curves post sterilisation was obtained using method described above. A comparison of pre and post sterilisation is shown in Fig. 7. As seen from the dose–response curves, the *I*_max_ values decrease post sterilisation as might be expected if there was lesser enzyme activity.

#### Initial in vivo studies with the microneedle array electrodes-based glucose biosensors

Studies over short time periods (ranging from 3 to 6 h) were done using sterilised microneedle array devices. These short-term studies were undertaken for the convenience of the volunteer as it involved mobilising next to the potentiostat setup. We anticipate that these studies will provide valuable feedback on the sensor and the supporting setup that will enable development of portable bio-instrumentation around the sensor for longer time measurements.

**Fig. 8:**
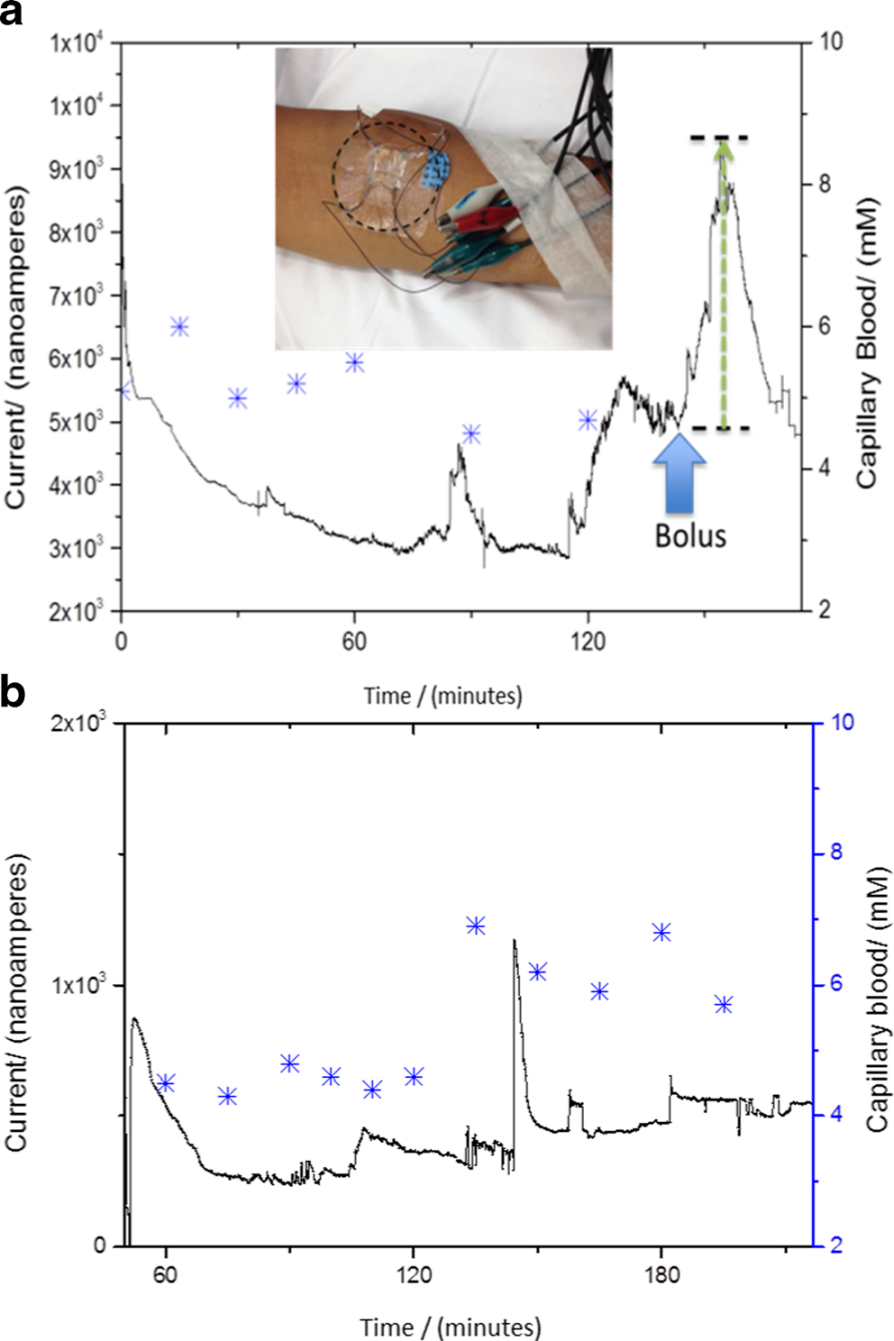
In vivo data obtained on two different days with two different devices after inserting the microneedle array electrodes on the forearm and biased at 0.7 V versus integrated Ag/AgCl reference electrode, plotted against finger stick (measurements represented as star sign); *inset*, showing the location on forearm where the devices were inserted for in vivo studies. **a** and **b** show in vivo measurements done over 3 h

Unprocessed data from our initial in vivo sensing after insertion of the device into the forearm of a healthy volunteer is presented here. As seen in Fig. 8, the sensors are measuring an output current from the skin ISF over 3–6 h. In both instances, it is evident that the sensor takes about an hour to reach a stable baseline. This could be the time required for the polyphenol films entrapped with the GOx enzyme to equilibrate with the environment within the dermal ISF. Many commercially available devices too have an equilibration time and therefore are recommended to be implanted a few hours before recording the glucose measurements [19].

As seen in Fig. 8a, the sensor shows an increased current value of 4500 nA in response to a glucose bolus (slice of cake in this instance) over duration of 45 min and drops back down. There is some correlation and time lag between the capillary blood and the dermal ISF (Fig. 8a, b). Whilst the dimensions are designed to obtain a minimally invasive sensor, it is evident from these in vivo studies that major operational challenge is to keep the sensors fixed in the sub-dermal space. As observed in Fig. 8b, artefacts can be seen due to movement of the arm. It is worth noting that the duration of these is much shorter than the duration of changes in glucose concentration.

There are various algorithmic approaches establishing the relationship between the measured current and glucose concentration; however, that is beyond the scope of this paper. For purposes of demonstrating the device function, we have presented unprocessed data. The sensor will be further optimised for in vivo measurements through clinical studies in healthy volunteers and subjects with T1D. For the clinical assessments, we will use the YSI measurements of venous blood, a gold standard, for the optimisation of the devices. In real life scenario, we anticipate the use of capillary blood measurements as means for calibration continuous glucose monitoring sensors.

The ability to constantly and accurately monitor blood glucose levels with a discrete device represents the top research priority for people with type 1 diabetes and specialist clinicians [20]. One of the major barriers to achieving these priorities is sensor accuracy [21]. Reduced sensor accuracy of existing electrochemical continuous glucose sensors is particularly evident in the hypoglycaemic range, resulting in either missed episode of hypoglycaemia or false hypoglycaemic alarms with subsequent reduced sensor reliability and patient satisfaction [22].

In recent years, there has been increasing interest in the use of microneedle technology for transdermal drug delivery [23] and for sensing of ISF analytes [24]. By virtue of their minimally invasive nature, use of microneedle arrays is associated with no or minimal pain, no bleeding, minimal skin reaction, rapid skin recovery and a reduced infection risk. Moreover, they provide the potential to improve sensor accuracy by providing a large surface area for the electrochemical reaction providing the potential to improve the signal/noise ratio with subsequent improvement in device sensitivity and accuracy.

The use of multiple simultaneous glucose sensors improves accuracy and precision of continuous glucose monitoring [25]. Partitioning the microneedle array into four individual 4 × 4 sub-arrays provides redundancy for technical failure of single sensors as a single malfunctioning sensor can be voted out on the basis of divergent results using conventional voting protocols.

## Conclusion

We have demonstrated that microneedle sensors reproducibly responded to changing glucose concentrations with linear responses seen in the physiological range (0–30 mM). There was also no crosstalk between the microneedle arrays, which indicates the effectiveness of redundancy of these devices for single analyte measurement or multiplexed measurement of analytes.

Sterilisation of the microneedle devices by gamma ray irradiation did reduce the response of the device but that the devices still perform adequately, post sterilisation. The sterilised devices were stable over several days of storage at ambient conditions. The in vivo studies in human volunteer yielding measurable currents and correlation to the capillary blood measurements are important observations in preparation for clinical studies to assess safety, efficacy and correlation of the current values with that of the glucose concentration in the ISF in human subjects.

Continuous glucose monitoring technology has emerged as an important tool for management of people with diabetes. However, widespread use of this technology can only be achieved if the CGM devices become more accurate, reliable, less invasive and cost-effective.

In this paper, we describe fabrication and evaluation of a novel glucose sensor based on microneedle technology. This sensor performs electrochemically post sterilisation and can measure current in vivo when inserted in the forearm. This robust innovation has the potential to address some of the important challenges facing existing CGM devices including invasiveness, pain, accuracy and cost.

## Abbreviations

CGM: Continuous glucose monitoring
ISF: Interstitial fluid
MICoMS: Minimally invasive continuous monitoring sensors
T1D: Type 1 diabetes

## Footnotes

1 Minimally Invasive Continuous Monitoring Sensor (MICoMS)
